# Combined Targeting of Mutant p53 and Jumonji Family Histone Demethylase Augments Therapeutic Efficacy of Radiation in H3K27M DIPG

**DOI:** 10.3390/ijms21020490

**Published:** 2020-01-13

**Authors:** Anatoly Nikolaev, John B. Fiveash, Eddy S. Yang

**Affiliations:** Department of Radiation Oncology, University of Alabama at Birmingham, Birmingham, AL 35249, USA

**Keywords:** H3K27M, DIPG, p53, APR-246, Jumonji family histone demethylases, GSK-J4, radiation, DNA damage repair, glutathione depletion, oxidative stress induction

## Abstract

Diffuse intrinsic pontine glioma (DIPG) is an aggressive pediatric brainstem tumor with a 5-year survival of <1%. Up to 80% of the DIPG tumors contain a specific K27M mutation in one of the two genes encoding histone H3 (H3K27M). Furthermore, p53 mutations found in >70–80% of H3K27M DIPG, and mutant p53 status is associated with a decreased response to radiation treatment and worse overall prognosis. Recent evidence indicates that H3K27M mutation disrupts tri-methylation at H3K27 leading to aberrant gene expression. Jumonji family histone demethylases collaborates with H3K27 mutation in DIPG by erasing H3K27 trimethylation and thus contributing to derepression of genes involved in tumorigenesis. Since the first line of treatment for pediatric DIPG is fractionated radiation, we investigated the effects of Jumonji demethylase inhibition with GSK-J4, and mutant p53 targeting/oxidative stress induction with APR-246, on radio-sensitization of human H3K27M DIPG cells. Both APR-246 and GSK-J4 displayed growth inhibitory effects as single agents in H3K27M DIPG cells. Furthermore, both of these agents elicited mild radiosensitizing effects in human DIPG cells (sensitizer enhancement ratios (SERs) of 1.12 and 1.35, respectively; *p* < 0.05). Strikingly, a combination of APR-246 and GSK-J4 displayed a significant enhancement of radiosensitization, with SER of 1.50 (*p* < 0.05) at sub-micro-molar concentrations of the drugs (0.5 μM). The molecular mechanism of the observed radiosensitization appears to involve DNA damage repair deficiency triggered by APR-246/GSK-J4, leading to the induction of apoptotic cell death. Thus, a therapeutic approach of combined targeting of mutant p53, oxidative stress induction, and Jumonji demethylase inhibition with radiation in DIPG warrants further investigation.

## 1. Introduction

Diffuse intrinsic pontine glioma (DIPG) is an aggressive pediatric brainstem tumor, which constitutes about 75% of all brainstem malignancies found in children [1,2]. Approximately 350–400 new cases of DIPG are diagnosed each year in the United States [1,2,3]. The tumor usually affects the children 3–10 years of age. The common presenting neurological symptoms include diplopia, ataxia, unilateral hemiparesis, and signs and symptoms of an increased intra-cranial pressure [1,2]. The symptoms are usually rapidly progressing, with medial survival of less than 12 months [1,2,3]. DIPG is usually diagnosed by MRI [4]. Radiographic features on MRI are enlarged pons which is heterogeneously enhanced on T2 sequence [4]. The biopsy is usually not necessary [5]. Despite a significant effort to improve the outcomes of DIPG, prognosis remains dismal [1,2,3,4,5,6]. The effective treatments for DIPG have been elusive [6]. Multiple clinical trials failed to demonstrate any benefit of various chemotherapy regimens, including etoposide, cisplatin, and temozolomide [6,7,8,9,10]. The first line treatment option for DIPG is radiation, which provides a temporary symptomatic relief, with a mild improvement of overall survival [6,11,12]. Neither hyper-fractionated, nor hypo-fractionated radiation treatment showed improvement in overall survival over conventional fractionation (54 Gy in 30 fractions) in clinical trials [6,11,12]. DIPG relapse inevitably occurs within 6 months after the initial radiation treatment. Re-irradiation used to treat the relapsed disease. Recently, a hypo-fractionated re-irradiation regimen was reported to offer a numerical overall survival advantage in recurrent DIPG (36% vs. 26% 1-year overall survival favoring hypo-fractionation) [13].

On the molecular level, the two most frequently mutated genes in DIPG are histone H3 and *TP53* [14]. Up to 80% of DIPG tumors contain a specific K27M mutation in one of two genes encoding histone H3 (H3K27M). 60–75% of H3K27M mutations occur in gene encoding histone variant H3.3 [14]. This clinically aggressive subtype of DIPG is associated with mutations in *TP53* gene in 60–80% of cases [14,15]. Recent molecular studies demonstrated that H3K27M mutation acts as a gain-of-function mutation in DIPG [15]. It had been shown to block the activity of the Enhancer of Zeste Homolog 2 (EZH2) histone methyl transferase, the catalytic subunit of the Polycomb Repressive Complex 2 (PRC2) [15]. H3K27M mutation disrupts tri-methylation at H3K27 leading to global hypo-methylation and aberrant de-repression of gene expression normally silenced by PRC2 [15]. Jumonji family histone demethylases are believed to collaborate with H3K27 mutation in DIPG by erasing the tri-methylation mark on H3K27 and thus contributing to de-repression of genes involved in tumorigenesis [16]. A specific inhibitor of Jumonji family histone demethylase GSK-J4 was recently reported to restore H3K27 tri-methylation patterns in human DIPG cells and improve survival of H3K27M mutant orthotopic xenograft brainstem tumor models [16,17]. APR-246 is a novel mutant p53-targeting and oxidative stress inducing drug candidate that had been shown to interact with a wide range of p53 mutant proteins, and additionally to deplete glutathione, and to inhibit thioredoxin reductase activity, thus leading to accumulation of the reactive oxygen species (ROS). APR-246 was shown to have synergistic effects on cell death when combined with DNA-damaging agents such as chemotherapy and radiation in various cancer cell lines expressing mutant p53 protein [18,19]. Clinical evaluation of APR-246 in p53 mutant myelodysplastic syndromes (MDS) revealed a dramatic 82% rate of complete response, leading to a fast track designation and an orphan drug designation of APR-246 by Food and Drug Administration (FDA) in April 2019 [19,20]. Given the important role of p53 tumor suppressor activities and H3K27 methylation in DNA damage response [21,22], we investigated the efficacy of mutant p53 targeting, oxidative stress induction, and Jumonji family histone demethylase JMJD3 inhibition combined with therapeutic radiation in human DIPG cells. Our hypothesis was that dual targeting of the proposed epigenetic mechanisms of disease pathogenesis mediated by H3K27M and TP53 mutations would sensitize DIPG cells to therapeutic radiation.

## 2. Results

### 2.1. Mutant p53 Targeting Elicits Growth Inhibitory and Radio-Sensitizing Effects in H3K27M DIPG Cells

**Figure 1:**
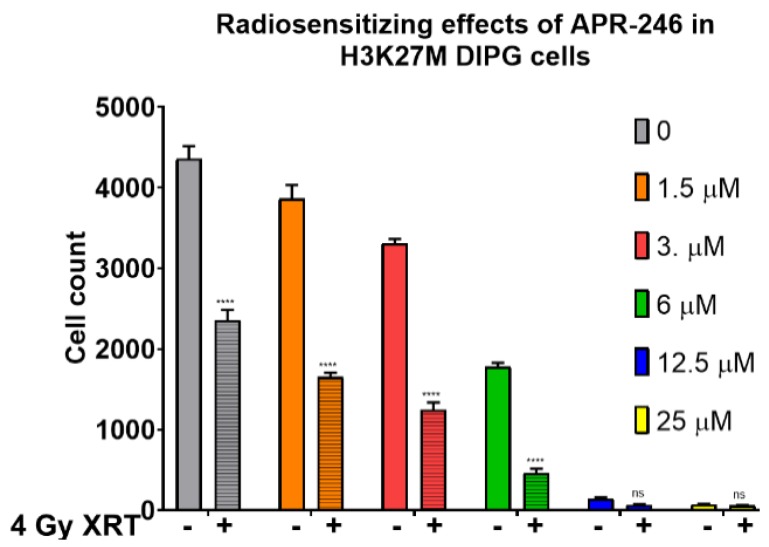
Anti-proliferative and radio-sensitizing effects of APR-246 in H3K27M DIPG cells. Proliferation assay with SF8628 pediatric diffuse intrinsic pontine glioma (DIPG) cell line harboring the histone H3.3 K27M mutation. The growth inhibitory and radiosensitizing effects of APR-246, by comparing the cell counts with 4 Gy dose (+) of radiation (crossed bars), versus without (−) radiation treatment (open bars). The cells were treated with the indicated concentrations of APR-246 for 6 h, then subjected to 4 Gy dose of radiation (or mock-treated). Ninety-six hours post-treatments, the cell counts were determined and plotted along the Y-axis. **** = *p* < 0.0001. ns = not statistically significant.

Since mutations in *TP53* gene are present in the majority of DIPG cases, and given the increased tumor aggressiveness and worse overall prognosis associated with *TP53* mutations DIPG, we investigated the efficacy of mutant p53 targeting with APR-246, a molecular agent shown to form covalent bonds with mutant p53 protein and to induce oxidative stress by glutathione depletion and thioredoxin reductase inactivation in a variety of cancer types [14,18]. Strikingly, we found that mutant p53 reactivating drug APR-246 elicited robust dose-dependent growth inhibitory effects as a single agent on H3K27M DIPG cells in proliferation assays (Figure S1). Furthermore, we found that mutant p53 targeting with APR-246 displays at least additive effects when combined with ionizing radiation in H3K27M DIPG cells, with 67% growth inhibition at 1.5 μM in combination with 4 Gy radiation dose (XRT) vs. 13% growth inhibition by APR-246 alone (*p* < 0.001) (Figure 1). The 4 Gy radiation dose is particularly relevant for hypo-fractionated re-irradiation regimens used to treat recurrent DIPG in clinic [13]. Importantly, we did not observe a significant effect of APR-246 in *TP53* null cells at these concentrations of the drug (Figure S2), indicating that the growth inhibitory and radio-sensitizing effects of APR-246 are dependent on the mutant p53 protein. 

**Figure 2:**
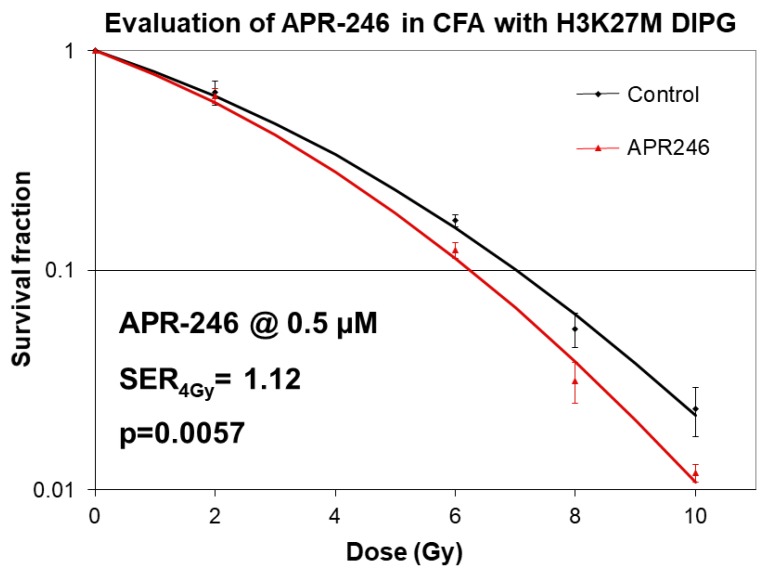
Radio-sensitizing effects of APR-246 in clonogenic survival assays in H3K27M DIPG cells. Clonogenic survival assays with SF8628 pediatric DIPG cell line harboring the histone H3.3 K27M mutation. DIPG cells were treated with 0.5 µM concentration of APR-246 both before and after radiation treatments (0–10 Gy doses). Survival fractions were determined 2 weeks after plating. Sensitizer enhancement rations (SERs) were calculated for the dose 4 Gy. Mean SERs from two independent experiments with 3 data points per experiment are shown followed by SER_4Gy_ = sensitizer enhancement ratio at 4 Gy radiation dose. Curve fitting for radiation dose response was performed using linear quadratic equation.

To determine whether APR-246 has a synergistic effect when combined with XRT, we carried out classical clonogenic survival assays, in which the concentration of the drug is held constant at 0.5 μM, while the radiation dose is varied from 0–10 Gy range [23]. Importantly, we found that APR-246 displayed significant radiation sensitizer effects at 4, 6, 8, and 10 Gy XRT (*p* < 0.01) at sub-micro-molar concentrations (0.5 μM) of the drug (Figure 2). The radiation SER was determined to be 1.12 (*p* < 0.01), indicating a statistically significant radio-sensitizing effect (Figure 2).

### 2.2. Inhibition of Jumonji Family Histone Demethylase with GSK-J4 Displays Anti-Proliferative and Radiosensitizing Effects on H3K27M Diffuse Intrinsic Pontine Glioma (DIPG) Cells

**Figure 3:**
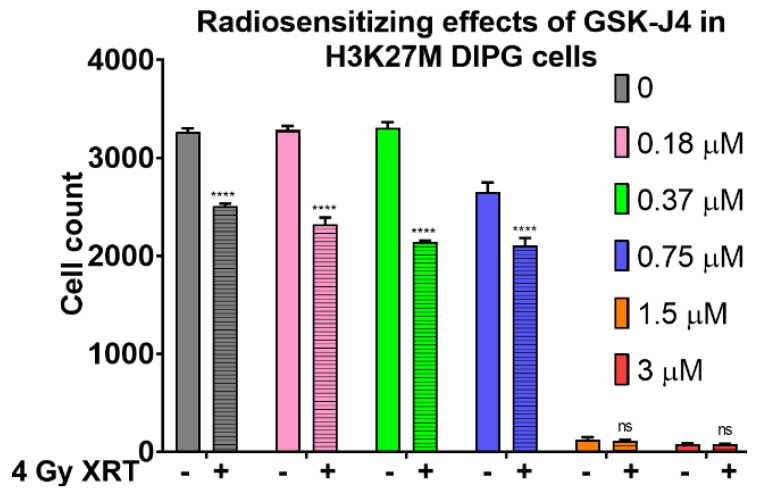
Growth inhibitory and radio-sensitizing effects of GSK-J4 in H3K27M DIPG cells in proliferation assays. Proliferation assay with SF8628 pediatric DIPG cell line harboring the histone H3.3 K27M mutation. The radio-sensitizing effects of GSK-G4 by comparing the cell counts with 4 Gy dose (+) of radiation (crossed bars), versus without radiation (−) treatment (open bars). The cells were treated with the indicated concentrations of GSK-J4 for 5 days to reverse the global hypomethylation of H3K27, then subjected to 4 Gy dose of radiation (or mock-treated). Ninety-six hours post-treatments, the cell counts were determined and plotted along the Y-axis. **** = *p* < 0.0001; ns = not statistically significant.

Since H3K27M mutation is known to abrogate EZH2-mediated silencing of genes involved in cell proliferation and tumorigenesis, and given the role of Jumonji family histone demethylase JMJD3 in exacerbating these effects of H3K27M mutation, we investigated the consequences of blocking Jumonji demethylase function with GSK-J4 inhibitor in DIPG cells. Consistent with the literature data, we found that GSK-J4 had a growth inhibitory effect on H3K27M mutant DIPG cells as a single agent, with 19% growth inhibition at 0.75 μM concentration, (*p* < 0.0001) (Figure S3). Since therapeutic radiation is the first line treatment that provides both symptomatic relief and survival advantage for DIPG patients, we investigated whether a combined approach of targeting H3K27M pathogenic mechanism with GSK-J4 inhibitor would enhance the growth inhibitory effects of radiation in DIPG cells. Importantly, we found that GSK-J4 inhibitor displayed at least additive radio-sensitizing effects when combined with ionizing radiation at sub-micro-molar concentrations of the drug, with 35% growth inhibition at 0.75 μM with 4 Gy XRT (*p* < 0.0001) (Figure 3). 

**Figure 4:**
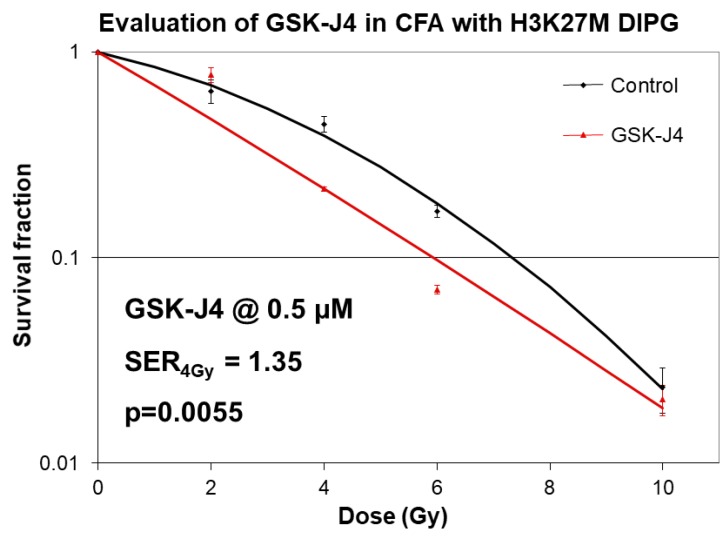
Radio-sensitizing effects of GSK-J4 in clonogenic survival assays in H3K27M DIPG cells. Clonogenic survival assays with SF8628 pediatric DIPG cell line harboring the histone H3.3 K27M mutation. DIPG cells were treated with 0.5 µM concentration of GSK-J4 both before and after radiation treatments (0–10 Gy doses). Survival fractions were determined 2 weeks after plating. Curve fitting was performed using linear quadratic equation. Sensitizer enhancement rations (SERs) were calculated for the dose 4 Gy. Mean SERs from two independent experiments with 3 data points per experiment are shown followed by SER_4Gy_ = sensitizer enhancement ratio at 4 Gy radiation dose. GSK-J4.

To determine whether the effects of GSK-J4 inhibitor are specific for H3K27M mutation, we evaluated the effects of this drug on H3K27 wild type cells (Figure S4). Intriguingly, we found that GSK-J4 displayed a significant growth inhibitory effect in H3K27 wild type cell lines, raising the possibility of H3K27M-independent effects of this drug. Furthermore, growth inhibitory effects of GSK-J4 appeared to be independent of TP53 status (Figure S4). Since radiation is the standard treatment modality for pediatric DIPG, and given the recently described important role of H3K27 tri-methylation in non-homologous end joining (NHEJ) type of DNA damage repair [22], we investigated the effects of GSK-J4 inhibitor combined with radiation in clonogenic survival assays (Figure 4). 

### 2.3. Combined Targeting of Mutant p53 and Jumonji Family Histone Improves Sensitivity of H3K27M DIPG Cells to Radiation

**Figure 5:**
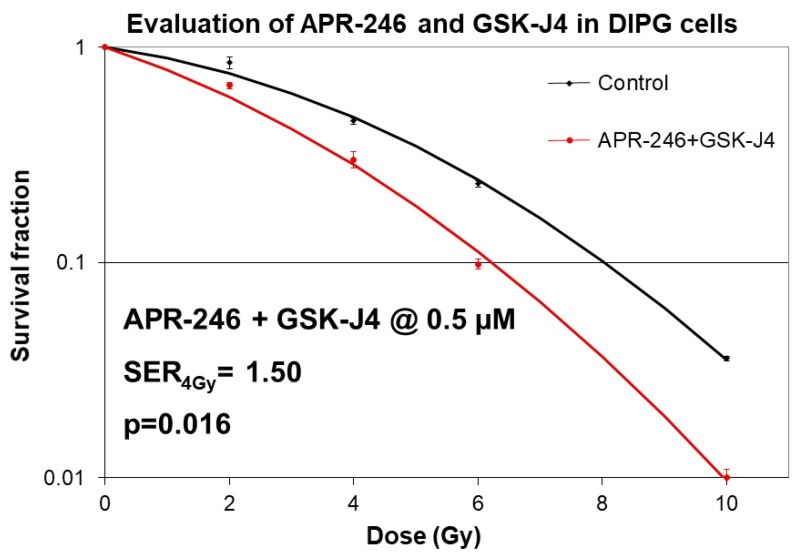
Radio-sensitizing effects of APR-246 combined with GSK-J4 in clonogenic survival assays in H3K27M DIPG cells. Clonogenic survival assays with SF8628 pediatric DIPG cell line harboring the histone H3.3 K27M mutation. DIPG cells were pre-treated for 5 days with 0.5 µM concentration of GSK-J4 and APR-246 before plating and radiation treatments (0–10 Gy doses). Survival fractions were determined 2 weeks after plating. Curve fitting was performed using linear quadratic equation. Sensitizer enhancement rations (SERs) were calculated for the dose 4 Gy. Mean SERs from two independent experiments with 3 data points per experiment are shown followed by SER_4Gy_ = sensitizer enhancement ratio at 4 Gy radiation dose.

Given the above described synergistic effects of Jumonji family histone demethylase inhibition and mutant p53 targeting on radiation sensitivities of H3K27M DIPG cells in clonogenic survival assays, we investigated the effects of dual inhibition of these molecular pathways on radio-sensitization. Strikingly, we observed a dramatic increase in radiation sensitivity when both pathways were targeted with molecular agents APR-246 and GSK-J4 (Figure 5). Specifically, we found that radiation SER is increased to 1.5 (*p* < 0.05) with combined APR-246 and GSK-J4 treatments, indicating that radiation treatment at 4 Gy dose is 50% more efficacious in killing H3K27M tumor cells at clinically relevant concentrations of the drugs (0.5 μM for both) (Figure 5).

### 2.4. Combined Inhibition of Jumonji Family Histone Demethylase with Mutant p53 Targeting and Oxidative Stress Induction Promotes Apoptosis of H3K27M DIPG Cells

**Figure 6:**
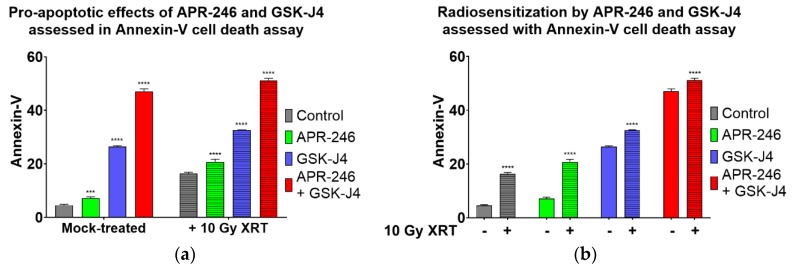
APR-246 combined with GSK-J4 induces apoptosis of H3.3 K27M DIPG cells following radiation treatment. Annexin V apoptosis assay with SF8628 H3.3 K27M DIPG cells. (**a**) Pro-apoptotic effects of APR-246 and GSK-J4 used as single agents (open bars on the left) or combined with XRT (crossed bars on the right), versus untreated cells; (**b**) Same experiment evaluating radiosensitization by comparing % Annexin-V cells treated with 10 Gy XRT vs. mock-treated cells. H3K27M DIPG cells were pre-treated with 5 µM of either APR-246, GSK-J4, or both, and subjected to a single 10 Gy dose of radiation. Cells were harvested 24 h post-irradiation and Annexin V cell death assay was carried out according to manufacturer’s protocols (Annexin V-FITC apoptosis kit, BioVision). **** = *p* < 0.0001. *** = *p* < 0.001; ns = not statistically significant.

To determine the consequences of the DNA DSBs repair deficiency elicited by GSK-J4 and APR-246, we investigated whether these agents would enhance the apoptotic cell death of H3K27M DIPG cells following radiation treatment (Figure 6). Intriguingly, both APR-246 and GSK-J4 potentiated apoptotic cell death, when used as single agent or in combination with radiation. Strikingly, a triple combination of APR-246, GSK-J4 and radiation resulted in the highest levels of apoptotic cell death in H3K27M DIPG cell (over 50%, Figure 6). 

### 2.5. Combined Targeting of H3K27M and Mutant p53 Driven Disease Mechanisms Induces a DNA Damage Repair Deficit Following Irradiation

**Figure 7:**
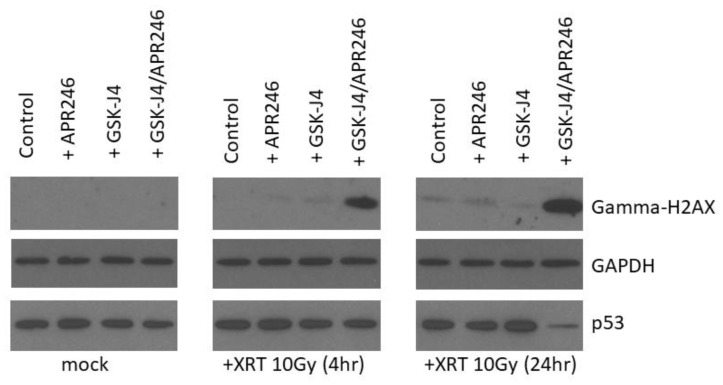
APR-246 combined with GSK-J4 induces DNA repair deficiency in H3.3 K27M DIPG cells following radiation treatment. Western blot analysis of SF8628 H3.3 K27M DIPG cell lysates with gamma-H2AX, p53, and GAPDH-specific antibodies. H3.3 K27M DIPG cells were pre-treated with 5 µM of either APR-246, GSK-J4, or both, and subjected to a single 10 Gy dose of radiation. Cells were harvested at the indicated time points (4 h and 24 h post-irradiation). Equal protein concentrations were loaded per each lane. Equal loading confirmed by GAPDH Western blotting.

To determine the molecular mechanism of GSK-J4 and APR-246-mediated radio-sensitization of H3K27M DIPG cells, we investigated the effects of these agents on the repair of DNA double strand breaks (DSBs) following radiation treatment (Figure 7). Neither GSK-J4 nor APR-246 were capable of producing a delay in double strand break repair, as assessed by the accumulation of gamma-H2AX 4 and 24 h following radiation treatment (Figure 7). In contrast, a combined dual inhibition of H3K27 demethylase by GSK-J4 and mutant p53 targeting by APR-246 produced a significant delay in double strand break repair, as evidenced by the accumulation of gamma-H2AX levels 4 and 24 h following radiation treatment (Figure 7). This result indicates that targeting of both H3K27M and mutant p53 pathogenic mechanisms produces a DNA DSB repair deficiency that contributes to enhanced radiation sensitivity of H3K27M DIPG cells and apoptotic cell death.

## 3. Discussion

Recent evidence indicates that H3 K27M mutation is the key epigenetic driver of the DIPG disease pathogenesis [13,14,15]. Indeed, molecular targeting of the H3 K27M epigenetic pathway with Jumonji family histone demethylase inhibitor GSK-J4 had been shown to decrease the viability of H3K27M tumor cells in vitro, and also reduced tumor growth in vivo in an orthotopic xenograft animal model. Furthermore, mutations in *TP53* tumor suppressor gene were recently found to be a major driver of DIPG radio-resistance in patients, as well as in DIPG cell models, derived from treatment-naïve stereotactic biopsies [24]. Strikingly, DIPG patients with *TP53* mutations were found to have a worse clinical and radiological response to radiation treatment compared to DIPG patients with wild-type *TP53*. *TP53* mutant patients also showed a shorter time to relapse after radiation treatment compared to the patients with wild-type *TP53*, and a worse overall prognosis [24]. Therefore, therapeutic targeting of mutant p53 is expected to improve the radiation sensitivities of DIPG tumors with mutant *TP53* status. Additionally, since the predominant mechanism of radiation-induced DNA damage is believed to be indirect, through generation of ROS (hydroxyl radicals), glutathione-depleting oxidative stress-inducing drugs such as APR-246 are expected to exacerbate cytotoxic effects of radiation in cancer cells. Indeed, our results clearly indicate that targeting of mutant p53 and induction of oxidative stress act synergistically to improve the therapeutic efficacy of radiation in clonogenic survival assays (Figure 2). Furthermore, when mutant p53 targeting/ROS induction is combined with an agent inhibiting H3K27 histone demethylase (GSK-J4), an additional enhancement of radiation sensitivity is observed (Figure 5). This result is in line with a recent study showing radio-sensitizing effects of GSK-J4 when used as a single agent [25]. To the best of our knowledge, our study is the first to utilize a dual targeted approach of mutant p53 reactivation/ROS induction combined with H3K27 demethylase inhibition in H3K27M DIPG cells, and the first to demonstrate an enhanced radio-sensitization of *TP53* mutant H3K27M DIPG cells with this dual targeting approach. The mechanism of the increased radiation sensitivity appears to involve a delay in DSBs repair following DNA damage by XRT, indicating that combined targeting of H3K27M and p53 mutations produces a DSB repair deficiency in DIPG cells (Figure 7). This DSBs repair defect likely causes further enhancement of radiation sensitivity, and translates into a significantly increased cell death following radiation treatment when both pharmacological agents are combined (Figure 5, Figure 6 and Figure 7). Interestingly, both mutant p53-targeting/ROS induction with APR-246 and H3K27 demethylase inhibition by GSK-J4 are able to elicit an apoptotic cell death even without radiation treatment, indicating that additional mechanisms such as activation of pro-apoptotic p53 target genes, depletion of glutathione, inactivation of thioredoxin reductase, and/or accumulation of reactive oxygen species, may contribute to the cytotoxicity elicited by these drugs. The efficacy of mutant p53 targeting combined with H3K27 histone demethylase inhibition in animal models of TP53 mutant DIPG warrants further investigation. If successful, such work may lead to the investigator-initiated clinical trials of targeted agents APR-246 and GSK-J4 combined with therapeutic radiation in DIPG patients. These targeted agents combined with radiation may be particularly useful for the treatment of the recurrent DIPG, which utilizes a hypo-fractionated radiation dose regimen, given the enhanced radio-sensitization observed at higher (hypofractionated) radiation doses [13]. In addition to the above described pathways, a genetic deletion of EZH2 had been recently shown to improve survival of a mouse neural stem cell of model of DIPG, suggesting that EZH2 may also be a potential therapeutic target [17]. Contrary to previously reported results with EZH2 inhibition in H3K27M model of DIPG, we found that EZH2 inhibitor had no effect on proliferation of human H3K27M DIPG cells as a single agent, or when combined with ionizing radiation (Figure S5). This result, however, appears to be consistent with the proposed mechanism of H3K27M pathogenesis [14,15]. Indeed, Dr. Allis laboratory demonstrated that H3K27M mutation acts as a gain-of-function mutation by inhibiting EZH2 activity in DIPG cells, thus promoting global demethylation and gene de-repression. Therefore, it seems plausible that further inhibition of EZH2 enzymatic activity with a small molecule inhibitor may actually exacerbate the pathogenic effects of H3K27M mutation. Several other therapeutic agents discovered serendipitously showed some initial promise in pediatric DIPG patients, but their exact mechanism of action is still evolving. ONC201, initially discovered as p53-independent inducer of TRAIL and a dopamine receptor D2 antagonist, gained a fast-track designation from FDA for the treatment of adult recurrent H3K27M mutant high-grade glioma, after it showed efficacy in an adult patient with H3.3 K27M glioblastoma in a Phase 2 trial [26]. However, further investigation into the mechanism of action of this drug revealed that ONC201 induces cell death by directly targeting a mitochondrial protease CIpP [27]. Nevertheless, a pediatric patient with H3K27M DIPG showed a good clinical and radiographic response to ONC201, and remains clinically improved for 22 months since the diagnosis, as per the recent case report [28]. A phase 1 clinical trial evaluating ONC201 in both newly diagnosed and relapsed/refractory H3 K27M DIPG is ongoing, with preliminary encouraging results [29]. Since ONC201 is believed to act independently of TP53 and H3K27M mutational status, presumably by targeting CIpP, further evaluation of triple drug combination is needed to assess for any synergy with therapeutic radiation treatment in H3K27M DIPG. 

## 4. Materials and Methods 

SF8628 pediatric DIPG cell line harboring the histone H3.3 K27M mutation was obtained from MilliporeSigma (Cat # SCC127, Burlington, MA, USA). UM-SSC1 and UM-SCC6 cell lines were obtained from University of Michigan. FaDu and Detroit cell lines were obtained from ATCC. APR-246 was obtained directly from APREA Inc. (Stockholm, Sweden). GSK-J4 inhibitor was obtained from Tocris (Cat # 4594, Bristol, UK). Gamma-H2AX, p53 (DO-1) and GAPDH-specific antibodies were obtained from Cell Signaling Technology (CST) (Danvers, MA, USA). Proliferation and clonogenic survival assays were performed as previously described [22] with modifications. For proliferation assays, DIPG cells harboring the histone H3.3 K27M mutation were pre-treated for 5 days with indicated concentrations of GSK-J4 (0–3 µM) or with APR-246 (0–25 µM), or EZH2 inhibitor EP005687 (0–3 µM), or mock-pretreated with DMSO, were then seeded in 24-well plates, at concentration 10,000 cells/well, then irradiated with 0 or 4 Gy dose of XRT, and harvested at 96 h after treatments. Cells were trypsinized, diluted 1:20 in isotonic saline solution, and counted using a Beckman Z1 Coulter particle counter. Cell counts were represented as cells/ml. For the clonogenic survival assays, H3K27M DIPG cells (SF8628) were pre-treated for 5 days with indicated concentrations of GSK-J4 (0–3 µM) or with APR-246 (0–25 µM), or EZH2 inhibitor EP005687 (0–3 µM), or mock-pretreated with DMSO as above, and plated at 125, 250, 500, or 1000 cells per 60 mm culture dish. Plated cells were then irradiated with 0, 2, 4, 8, or 10 Gy XRT and remained undistributed for two weeks. Cells were fixed and stained, and the number of colonies were counted. Survival fraction was calculated by taking a ratio of (# colonies in experimental plate/# cells seeded in experimental plate)/(plating efficiency in control plate). Linear quadratic equation was used for survival curve fitting. Sensitizer enhancement ratios (SERs) was determined at 4Gy dose as indicated in figure legends. Experiments were performed at least in triplicate. Apoptosis was analyzed using the Annexin V-FITC kit (BioVision, Milpitas, CA, USA) according to the manufacturer′s instructions and as previously described [23].

## 5. Conclusions

Dual targeting of the two most prevalent pathogenic mutations in DIPG, H3K27M, and *TP53*, with pharmacological agents GSK-J4 and APR-246, sensitized H3K27M DIPG cells to radiation by producing DNA damage repair deficiency and enhancing apoptotic cell death. Therapeutic efficacy of mutant p53 targeting and Jumonji family demethylase inhibition combined with radiation in animal models of H3K27M DIPG warrants further investigation. 

## Supplementary Materials

Supplementary materials can be found at https://www.mdpi.com/1422-0067/21/2/490/s1.

## Abbreviations

H3K27M	Histone H3 Lysine 27 to Methionine Mutation
DIPG	Diffuse Intrinsic Pontine Glioma
DSBs	Double Strand Breaks
CD	Calculated Dose
XRT	Ionizing radiation treatment
APR-246	Mutant p53-reactivating agent
GSK-J4	Jumonji family H3K27 histone demethylase inhibitor
SER	Sensitizer Enhancement Ratio
ROS	Reactive oxygen species
