# Supplementary material for: Combined Targeting of Mutant p53 and Jumonji Family Histone Demethylase Augments Therapeutic Efficacy of Radiation in H3K27M DIPG
Source: Int J Mol Sci. 2020 Jan 13;21(2):490. doi: 10.3390/ijms21020490 (PMC7014308; doi:10.3390/ijms21020490)
Supplement: Supplementary file 1 [file ijms-21-00490-s001.zip › ijms-673615-supplementary.docx]

Supplementary


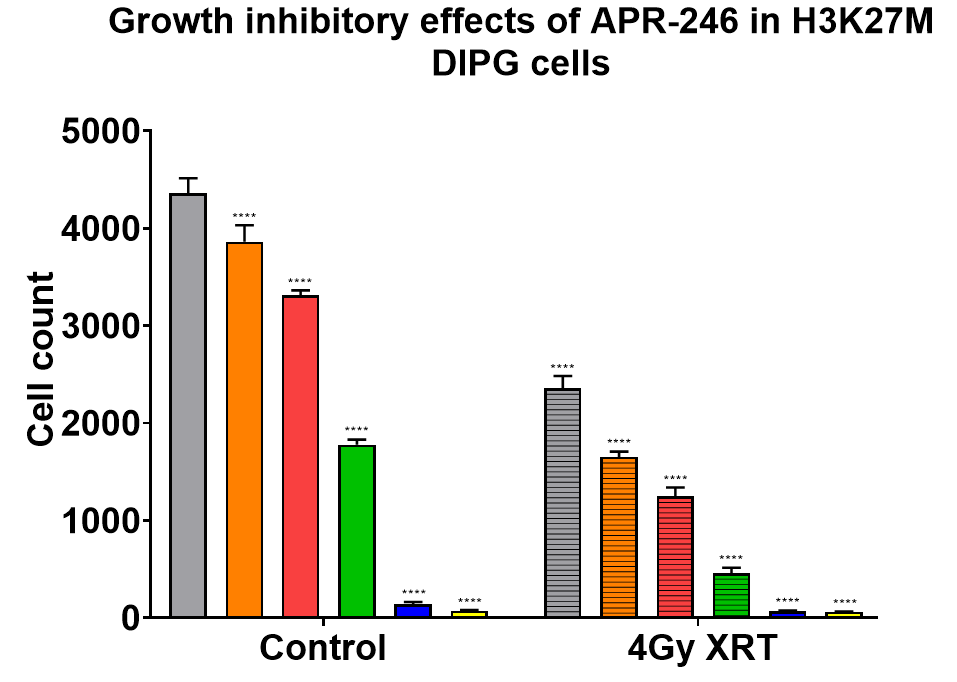


**Supplementary Figure 1.** Evaluation of the anti-proliferative and radio-sensitizing effects of APR-246 in H3K27M Diffuse intrinsic pontine glioma (DIPG) cells. Dose-dependent growth inhibitory effects of APR-246 as a single agent (open bars on the left) or in combination with radiation (crossed bars on the right). The cells were treated with the indicated concentrations of APR-246 for 6 h, then subjected to 4 Gy dose of radiation (or mock-treated). Ninety-six hours post-treatments, the cell counts were determined and plotted along the Y-axis. **** = *p* < 0.0001. ns = not statistically significant.


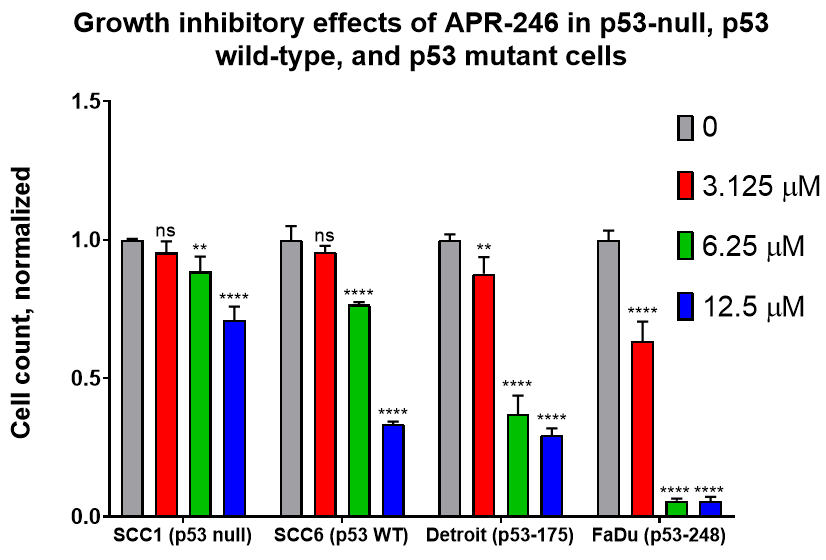


**Supplementary Figure 2.** Evaluation of mutant p53-reactivating/ROS inducing agent APR-246 in proliferation assay with p53-null SCC-1 cells, p53-wild-type SCC6 cells, and p53 mutant Detroit and FaDu cells. APR-246 selectivity towards mutant p53 expressing cells was evaluated in proliferation assays as described in Materials and Methods.


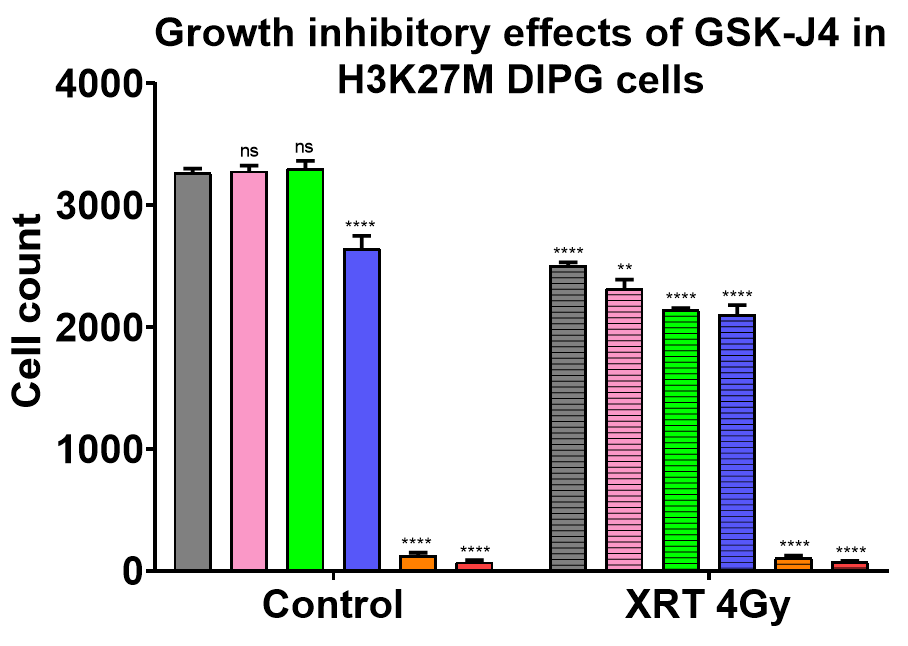


**Supplementary Figure 3.** Anti-proliferative effects of GSK-J4 in H3K27M DIPG. Proliferation assay with SF8628 pediatric DIPG cell line harboring the histone H3.3 K27M mutation. Dose-dependent growth inhibitory effects of GSK-J4 as a single agent (open bars on the left) or in combination with radiation (crossed bars on the right). The cells were treated with the indicated concentrations of GSK-J4 for 5 days, then subjected to 4 Gy dose of radiation (or mock-treated). Ninety-six hours post-treatments, the cell counts were determined and plotted along the Y-axis. **** = *p* < 0.0001. *** = *p* < 0.001; ** = *p* < 0.01; ns = not statistically significant.

| 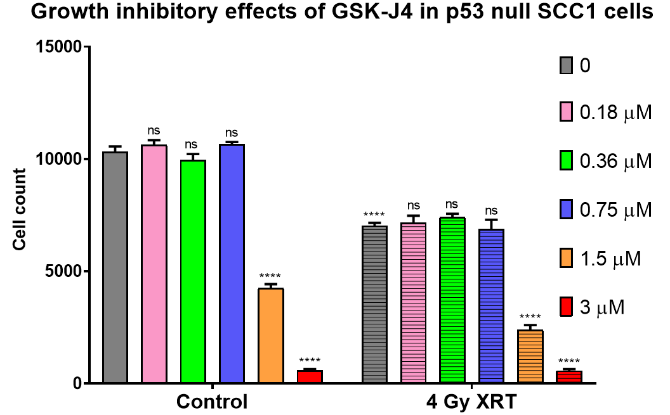 | 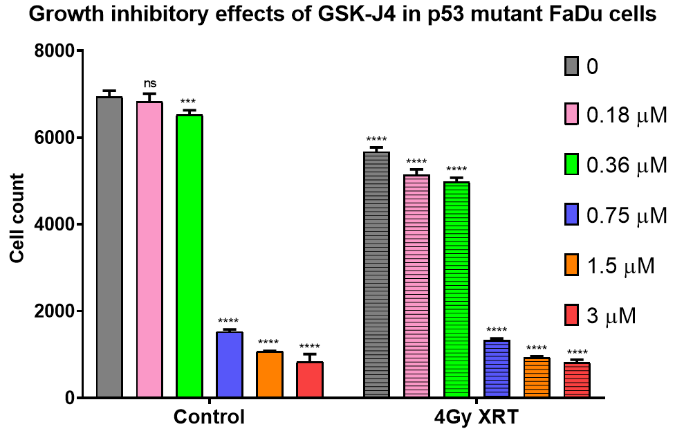 |
| --- | --- |
| (**a**) | (**b**) |

**Supplementary Figure 4.** Evaluation of Jumonji family histone demethylase inhibitor GSK-J4 in proliferation assay with H3K27-wild-type cells (p53-null SCC-1 cells, p53-wild-type SCC6 cells). GSK-J4 selectivity toward the H3K27-wild-type cells was evaluated in proliferation assays as described in Materials and Methods. (**a**) Dose-response with GSK-J4 as single agent or combined with XRT in proliferation assay with H3K27-wild-type p53-null SCC1 cells; (**b**) Dose-response with GSK-J4 as single agent or combined with XRT in proliferation assay with H3K27-wild-type p53-mutant FaDu cells.


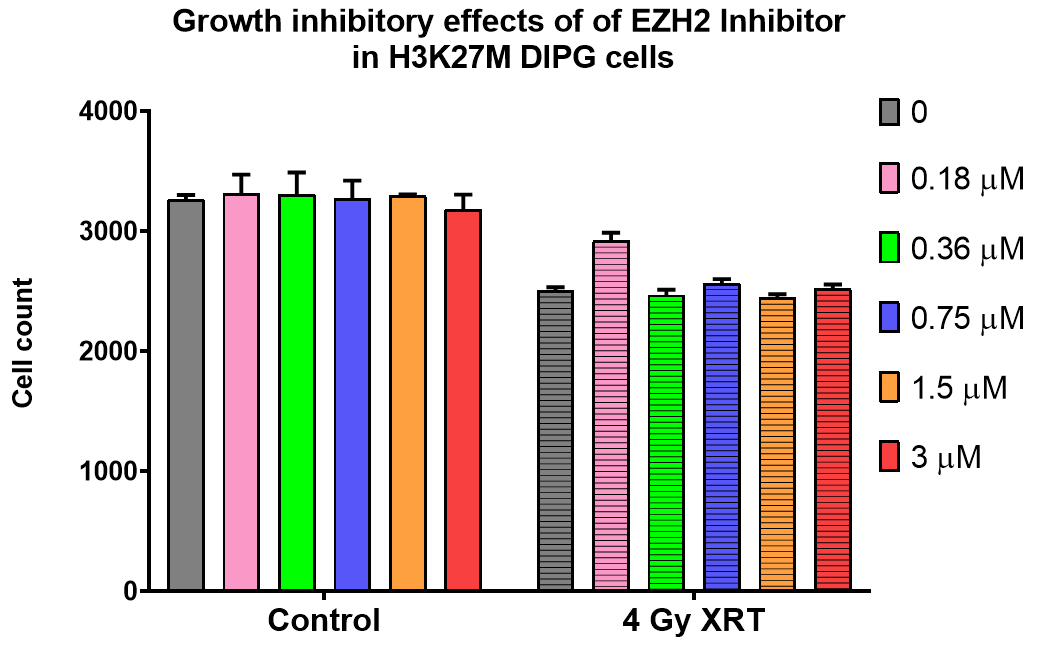


**Supplementary Figure 5.** The effects of EZH2 inhibitor EP005687 on proliferation and radiation sensitivities of H3K27M DIPG. Proliferation assay with SF8628 pediatric DIPG cell line harboring the histone H3.3 K27M mutation. The cells were pre-treated with the indicated concentrations of EZH2 inhibitor EP005687 for 5 days, then subjected to 4 Gy dose of radiation (or mock-treated). Ninety-six hours post-treatments, the cell counts were determined and plotted along the Y-axis. Open bars: mock radiation treatments (0 Gy). Horizontally crossed bars: 4 Gy radiation treatments.
